# The environmental health impacts of Russia’s war on Ukraine

**DOI:** 10.1186/s12995-023-00398-y

**Published:** 2024-01-05

**Authors:** Daniel Hryhorczuk, Barry S. Levy, Mykola Prodanchuk, Oleksandr Kravchuk, Nataliia Bubalo, Alex Hryhorczuk, Timothy B. Erickson

**Affiliations:** 1 Divisions of Environmental and Occupational Health Sciences and Epidemiology, University of Illinois School of Public Health, Chicago, USA; 2 Department of Public Health and Community Medicine, Tufts University School of Medicine, Boston, USA; 3 L.I.Medved’s Research Center of Preventive Toxicology, Food and Chemical Safety, Ministry of Health, Kiev, Ukraine; 4 Third Horizon Strategies, Chicago, USA; 5 Department of Emergency Medicine, Division of Medical Toxicology, Mass General Brigham, Harvard Medical School, Harvard Humanitarian Initiative, Boston, USA

**Keywords:** Environment, War, Public health, Ukraine, Russia, Ecocide, Human rights, International humanitarian law, War crimes

## Abstract

**Background:**

Russia’s invasion of Ukraine in February 2022 ignited the largest armed conflict in Europe since World War II. Ukrainian government agencies, civil society organizations, and international agencies have gathered an unprecedented amount of data about the impact of war on the environment, which is often the silent victim of war. We review these data and highlight the limitations of international governance for protection of the environment during time of war.

**Methods:**

We performed an integrative review of academic, institutional, and media information resources using the search terms “Ukraine”, “Russia”, “war”, “environment”, “health”, “human rights”, “international humanitarian law”, “international human rights law”, “ecocide”, and “war crimes”.

**Main text:**

Nearly 500,000 military personnel have been killed or wounded during the war, and more than 30,000 civilians have been killed or injured. Indirect health effects of the war have likely accounted for an even greater amount of civilian morbidity and mortality. The war has displaced more than 11 million people. Russia’s military forces have caused extensive damage to civilian infrastructure. The war has devastated Ukraine’s economy and reduced food and energy security in many countries.

The war has caused more than $56.4 billion in damage to the environment. There has been widespread chemical contamination of air, water, and soil, and 30% of Ukraine has been contaminated with landmines and unexploded ordnance. Landscape destruction, shelling, wildfires, deforestation, and pollution have adversely affected 30% of Ukraine’s protected areas. Russia’s seizure of the Zaporizhzhia Nuclear Power Plant and destruction of the Nova Kakhovka Dam have posed risks of long-term environmental catastrophe. Most of these environmental impacts threaten human health.

**Conclusion:**

In addition to enormous human costs, Russia’s war on Ukraine has had devastating impacts on the natural environment and the built environment. International law mandates that methods of warfare must be implemented with due regard to the protection and preservation of the natural environment. A just and lasting peace necessitates, among other requirements, rebuilding and restoration of Ukraine’s natural environment and built environment. The environmental consequences of all wars need to be investigated and more effective measures need to be implemented to protect the environment during war.

## Background

On February 24, 2022, Russia’s invasion of Ukraine launched the largest armed conflict in Europe since the World War II. While the effects of war are generally calculated in terms of human, economic, and social costs, the environment is often the silent victim of war. Environmental damage resulting from war can be both an unintended consequence of military activity and part of a military strategy. The environment can suffer collateral damage from military actions, such as shelling leading to wildfires; deliberate damage from “scorched earth” tactics, such as flooding from the destruction of dams; defensive tactics, such as digging trenches and laying antitank mines; and military activities that are conducted in environmentally sensitive areas, such as nature reserves. Both Russia’s offensive and Ukraine’s defensive military actions have adversely impacted the environment. Ukrainian government agencies, civil society organizations, and international agencies have gathered an unprecedented amount of data about the environmental impacts of the war. We review these data and highlight the limitations of international governance for protection of the environment during armed conflict and for accountability for those directly responsible for environmental damage.

## Methods

We performed an integrative review of academic, institutional, and media information resources on the environmental health impacts of Russia’s war in Ukraine. We conducted a search for combinations of the search terms “Ukraine”, “Russia”, “war”, “environment”, “health”, “human rights”, and “international humanitarian law” on PubMed and Google. We also searched the HeinOnline Law Journal Library and EJIL:Talk! Blog of the European Journal of International Law, using combinations of the search terms “ecocide”, “international humanitarian law”, “environment”, “international human rights law”, and “war crimes”. We limited searches to articles in English and Ukrainian. We then reviewed the titles of the identified articles for topical relevance. In addition, we drew on our participation in the NATO Science and Peace for Security Advanced Research Workshop on Health and Environment in Conflict Zones (SPS.ARW.G5432), held in Kyiv, Ukraine, in 2018 (DH, MP, OK, NB, AH, TE) and in the NATO Science and Peace for Security Advanced Research Workshop on Chemical, Biologic, Radiologic, and Nuclear Agents (SPS.ATC.G5663), held in Dnipro, Ukraine, in 2019 (DH, MP, OK, TE).

## Main text

By October 2023, nearly 500,000 Ukrainian and Russian military personnel had been killed or wounded during the war [1], and 30,000 civilians had been killed or injured, according to the Office of the High Commissioner on Human Rights [2]. In addition, indirect health effects of the war have likely accounted for an even greater amount of civilian morbidity and mortality due to malnutrition, communicable diseases, exacerbation of noncommunicable diseases, maternal and infant disorders, and mental and behavioral disorders. These indirect health effects of war are primarily caused by forced displacement and damage to civilian infrastructure. In November 2023, UNHCR: The United Nations Refugee Agency reported that nearly 6.3 million Ukrainians had become refugees, 5.0 million had been internally displaced, and over 17 million needed humanitarian assistance [3]. Russia’s military forces have caused extensive damage to civilian infrastructure for healthcare, agriculture and food supply, water and sanitation, energy, transportation, and communication. In addition, the war has had a devastating impact on Ukraine’s economy and has adversely affected food and energy security in many countries.

The Kyiv School of Economics estimated that, as of April 2023, the direct documented damages to Ukraine’s infrastructure were $147.5 billion (at replacement cost). The damaged infrastructure included 158,000 damaged or destroyed residential buildings, over 3,200 educational facilities, and 806 healthcare facilities in addition to damage to public infrastructure and industrial and agricultural assets [4]. The war has had a devastating economic impact on Ukraine; in 2022 alone, its gross domestic product (GDP) contracted by about 30% [5] and Ukraine is now the poorest country in Europe with a Gross National Income (GNI) per capita of only $3,500 [6].

The geopolitical, economic, and social impacts of the war go well beyond Ukraine.

Because Ukraine and Russia are two major suppliers of energy, food, and fertilizer, the war has had a profound impact on global food security, causing disruptions in supply chains and raising prices for these commodities, thereby exacerbating food shortages and inflation in many countries [7, 8]. Partially due to the war, global economic growth slowed to 3.2% in 2022, well below expectations [9]. The war has led to a significant energy crisis in Europe, with global reverberations [10].

Ukraine, which has been called “the breadbasket of Europe”, is a leader in food production and export, helping to feed 400 million people outside of Ukraine. In 2021–2022, it accounted for 41% of sunflower oil, 17% of barley, 13% of corn, and 9% of wheat production and export globally [11, 12]. Some effects of the food crisis were temporarily mitigated by a UN-brokered grain deal (Black Sea Agreement), but on July 17, 2023, Russia announced its withdrawal from the agreement and then bombarded Ukraine’s Black Sea ports and grain terminals, thereby restricting Ukraine’s export of grain [13].

The war has had devastating impacts on Ukraine’s natural environment and built environment [14–16]. The most striking example has been the destruction of the Nova Kakhovka Dam, as described below [17]. The adverse impacts on land-based and aquatic ecosystems have had detrimental effects on human health and well-being. These natural ecosystems perform fundamental life-support services upon which human health and well-being depend, such as provision of food, fiber, and fuel. These ecosystems also regulate air quality and climate; water purification; support of ecosystem function, such as soil formation and nutrient recycling; and provision of cultural services, such as spiritual enrichment, artistic inspiration, and recreation [18, 19]. Ukrainian and international environmental agencies as well as nongovernmental organizations have been documenting the environmental damage caused by the war, with the goal of seeking reparation and restoration of both the natural environment and the built environment during the postwar reconstruction period [11].

### Air pollution

The impacts of the war on air quality in Ukraine have been complex, dynamic, and have included both increases and decreases in air pollution.

#### Increases in air pollution

Major increases in air pollution have resulted from the following:

- Bombing: The concentration of fine particulate matter has dramatically increased because of bombing and resultant structural fires [20]. For example, in Kyiv, the concentration of fine particulate matter on March 19, 2022 – less than 1 month after the start of Russia’s invasion – was 27.8 times the World Health Organization recommended guideline.
- Destruction of fuel storage facilities: During the first 13 months of war, 36 fuel storage facilities were destroyed, including 17 oil depots, thereby generating pollutants from the burning of 108,000 tons of oil, oil products, and gasoline [11].
- Attacks on industrial facilities: These attacks, such as on fertilizer and nitric acid plants, have resulted in release of toxic substances, including nitric acid and ammonia [21].
- Movement of military equipment: Large-scale movement of military equipment, including tanks, artillery, armored vehicles, and trucks, has generated large amounts of dust as well as fossil-fuel emissions. These greenhouse gas emissions further contribute to global warming.
- Building destruction: Destruction of residential and other buildings has resulted in exposures to the toxic residues from explosions as well as hazardous dust and toxic substances, such as alkaline dust, cement particles, glass, asbestos, lead and other heavy metals, and organic substances, including polycyclic aromatic hydrocarbons [21, 22].
- Wildfires: Wildfires have occurred frequently and spread extensively because of military operations and an inadequate number of firefighters. In 2022, there were 25 times more forest fires than in 2021 [23]. Over183,000 hectares of Ukraine’s forests and plantations have been burned by wildfires [11]. Smoke from wildfires contains fine and coarse particulate matter, carbon monoxide, methane, oxides of nitrogen, volatile organic compounds, and many other toxic substances [24].

#### Decreases in air pollution

The emissions of several priority pollutants, such as nitrogen dioxide and sulfur dioxide, have decreased due to the negative impacts of the war on Ukraine’s economy, with resultant reduction in anthropogenic emissions from the closure of factories and construction sites as well as decreased civilian use of vehicles. During the first 2 weeks of the war, satellite images demonstrated reductions in atmospheric concentrations of nitrogen dioxide, fine particulate matter, and carbon monoxide [25, 26]. As the war evolved, the atmospheric concentration of nitrogen dioxide continued to be reduced and the airborne level of sulfur dioxide, which had increased during the first 2 weeks of the war, decreased.

#### Chemical weapons

Russia’s full-scale war against Ukraine has raised the threat of the use of chemical weapons. Ukraine has accused Russia of use of riot control agents and white phosphorus bombs, although these reports have not been confirmed due to the difficulties of on-site verification by the inspector of the Organization for the Prohibition of Chemical Weapons (OPCW) during active combat [27–29].

The war has exposed segments of the Ukrainian population to complex mixtures of toxicants. While it has not yet been possible to perform a complete investigation of related health effects, once the war is over, it will be necessary to treat and rehabilitate thousands of people whose health has been adversely affected by environmental toxicants and psychological stress.

#### Impacts on climate change

The war has increased Ukraine’s vulnerability to climate change and complicated its efforts to reduce greenhouse gas emissions [23]. During the first 12 months of the war, an estimated 21.9 million tons of carbon dioxide equivalents (tCO_2_e) were released due to war-related activities and an additional 17.7 million tCO_2_e were released from war-related fires [30].

Before the war, Ukraine’s goal was to reduce its energy use by two-thirds. Nationwide, installed renewable energy then was about 10 gigawatts -- more than 13% of all installed energy in Ukraine [31]. Russia’s weaponization of energy supplies, destruction of Ukraine’s power generation and heating infrastructure, widespread deforestation, and damage to Ukraine’s renewable energy systems has made this goal much more difficult to achieve. Due to damage to substations and networks, shelling, theft of equipment by occupiers, and lack of access to power plants, development of renewable energy in the temporarily occupied territories stopped. By the end of October 2022, about 75% of wind stations and nearly 50% of solar stations were decommissioned, mostly in the south of Ukraine [31]. Funds that could have been used to address climate change have been re-directed to respond to the consequences of war. As a result, the war will adversely affect the net-zero pledges of many countries, likely worsening the climate crisis and delaying the global transition to renewable energy [23].

### Water pollution and related issues

Military operations cause chemical pollution of freshwater resources directly – from dumping of ammunition and war equipment, decomposition of ammunition, and release and leaching of explosive residues – and indirectly from damage to industrial facilities [32]. Groundwater, which meets 25% of Ukraine’s drinking water needs, can be contaminated by leaching of explosive residues, such as perchlorate and nitrate, from soil [33, 34]. During war, damage to water infrastructure occurs directly and indirectly as a result of military attacks, despite international conventions that ban attacks on water infrastructure (such as dams) when the civilian damage is out of proportion to military advantage. These attacks deprive people of drinking water, disrupt sanitation, and pollute surface water and groundwater.

Before the war, Ukraine had a highly developed water sector, which has since been devastated by Russia’s invasion. As of July 2023, Ukraine’s Ministry of Environmental Protection and Natural Resources had documented the destruction of 724 hydraulic structures, 71 water pumping stations, 64 sewage pumping stations, and 23 water treatment facilities [35]. As a result of the war, 20.7 billion cubic meters of wastewater have been discharged into surface waters. In April 2022, about 6 million people in Ukraine – about 15% of the population – had limited or no access to safe water [32]. After the destruction of the Nova Kakhovka Dam in June 2023, approximately 1.25 million people and over 300,00 children in the Dnipro, Zaporizhzhia, Mykolaiv, and Kherson oblasts were without stable and safe drinking water supplies [36].

The flooding of abandoned coal mines has also posed risks for contamination of surface water and groundwater. When a mine ceases to operate, water must be pumped out of the underground shafts to prevent them from flooding. In 2019, the runoff of contaminated water from flooded mines in eastern Ukraine, totaling 760 million cubic meters, deposited almost 2.5 million tons of salts and other contaminants into the Severniy Donets River and the Sea of Azov [37]. As of July 2023, over 49 mines had been flooded in territories in eastern Ukraine occupied by Russian forces. The most serious threats have been from the Oleksandr-Zakhid mine, in which chlorobenzene and other hazardous wastes have been stored since 1989, and the Yunyi Komunar mine, in which the Soviet Union detonated a 0.3-kiloton nuclear bomb in 1979 to facilitate release of methane [38]. The Yunyi Komunar mine was closed in 2002 and pumping operations ceased in 2018, increasing the risk that water containing radioactive isotopes could spill and contaminate surrounding soil, rivers, ground water and threaten drinking water supplies.

**Fig. 1:**
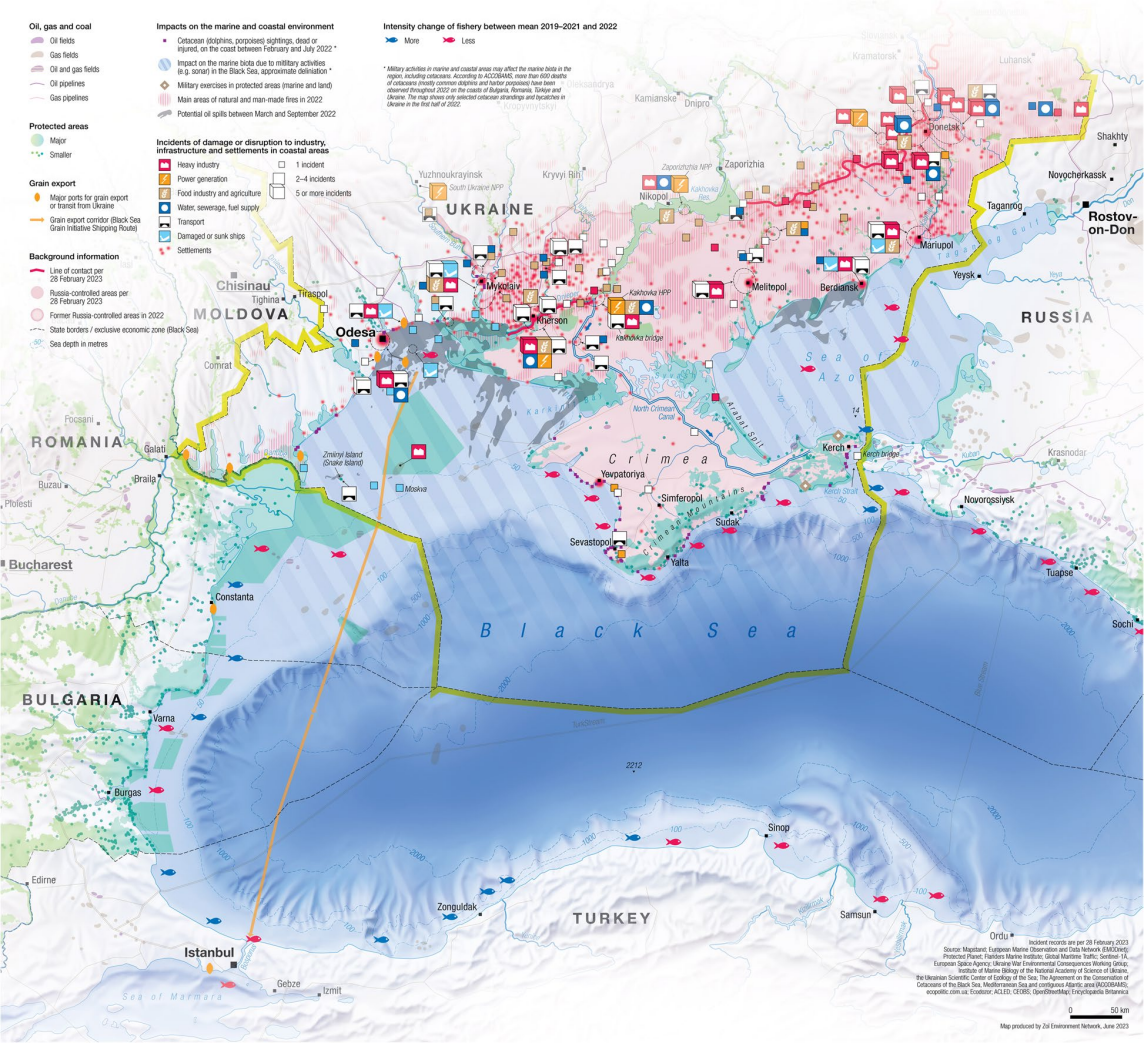
Ukraine conflict marine and coastal environmental threats. https://ceobs.org/ukraine-conflict-environmental-briefing-the-coastal-and-marine-environment/

Ukraine has about 2,700 km (1,674 miles) of coastline along the Black Sea and the Sea of Azov. Major ports, such as Odesa, Mykolayiv, and Mariupol, have been targets of repeated and prolonged attacks by Russia. These attacks and others along rivers, estuaries, and the Black Sea and Azov Sea coastlines have caused oil spills and other incidents of water pollution. Russian forces have deprived Ukraine’s access to ports and fishing resources in Crimea and Sea of Azov, resulting in a 67% decline in fish catch [35]. Before the war, both the Black Sea and Sea of Azov suffered decades of environmental degradation. The Black Sea, which receives the outflow of Europe’s three largest rivers, has a catchment area that is approximately five times greater than its surface area. Because this area includes major industrial facilities and vast agricultural lands, water pollution has been prevalent. The Black Sea’s great depth and shallow outlet result in little water mixing, and below 100 m it is largely devoid of oxygen. The Sea of Azov is extremely shallow and dominated by the inflow of the Don and Kuban rivers. The nutrients contained in its shallow waters once supported high fish stocks; however, eutrophication, pollution, overfishing, and coast military activity have degraded its ecosystems (Fig. 1) [33].

Primary impacts of the war on coastal and marine ecosystems have included chemical pollution, loud noise, physical damage to habitats from shelling and fortifications, and curtailment of conservation activities [33]. Landmines have been deployed on beaches and other coastal areas to prevent amphibious landings. In addition, both Russia and Ukraine have deployed sea mines. Sonar systems used by navies for detecting underwater vessels in the Black Sea have been associated with dolphin strandings. Major rivers, such as the Dnipro, Dneister, and Don, which drain into the Black Sea and the Sea of Azov, have carried toxic substances from land-based military activities. The collapse of the Nova Kakhovka Dam on the Dnipro River on June 6, 2023, caused massive flooding downstream, which carried organic wastes, hundreds of tons of oil, landmines, and unexploded ordnance into the Dnipro River Delta and the Black Sea.

### Soil contamination

#### Physical disturbances

Physical disturbances include excavation of tunnels and trenches, compaction by large-scale movements of troops with machinery, and cratering by explosives. The Russian fortifications in Ukraine are the most extensive defensive works since World War II. They span over 1,000 km (about 620 miles) and include anti-tank ditches, trenches, and concrete barriers. Tens of thousands of artillery rounds are being fired daily, pockmarking the land with craters.

#### Chemical contamination

Chemical contamination occurs through use of munitions, chemical spills from damage to industrial facilities and waste sites, and leaks and spills of oils and lubricants. Chemicals, especially non-biodegradable elements and compounds used in military ammunition and explosives, may contaminate soil and surface waters and may later adversely affect human and ecosystem health. Heavy metals are among the most frequent and most persistent contaminants in war zones, including lead, antimony, chromium, arsenic, mercury, nickel, zinc, cadmium, and copper. For example, soils of the Flanders region of Belgium during World War I still contain elevated concentrations of copper due to the intense shelling on battlefields there over 100 years ago. In France, an exclusion area known as “Zone Rouge” remains too disrupted for farming because of *bomburdation* – littering of a former battlefield with remnants of military debris, unexploded shells, and munitions [39]. Materials in weapons systems, which comprise both explosives and propellants, also contaminate the soil. Explosives most often used include hexahydro-1,3,5-trinitro-1,3,5-triazine (RDX), 2,4,6-trinitrotoluene (TNT), and octahydro-1,3,5,7-tetranitro-1,3,5,7-tetrazocine (HMX). Less commonly, explosives include nitroglycerin, 1,3,5-trinitrobenzene (TNB), dinitrobenzene (DNB), 2,4,6-trinitrophenol, and N-methyl-N,2,4,6-tetranitroaniline (tetryl), nitroguanidine (NQ), nitrocellulose (NC), 2,4- dinitrotoluene (2,4-DNT), and perchlorate [40, 41]. The environmental fates of these energetic materials are highly variable, dependent on their specific physiochemical properties and biodegradability. Munitions may also contain per- and polyfluoroalkyl substances (PFAS), which persist in the environment for long periods [42].

#### Landmines and unexploded ordnance

As a result of Russian aggression, Ukraine is heavily contaminated with landmines and unexploded ordnance [43]. During much of the war, tens of thousands of artillery shells have been fired in Ukraine daily. As of April 2023, approximately 174,000 square kilometers of Ukrainian territory (29%) were contaminated with landmines, deployment of which has been documented in 11 of Ukraine’s 27 regions. Minefields, with both anti-vehicle and anti-personnel landmines, extend for much of the 2,500 km (1,550-mile) contact line between the territories controlled by Ukraine and Russian forces. In addition to military casualties, between February 2022 and May 2023, a total of 855 civilians were reported as injured or killed in 550 mine-related incidents [44]. Tragically, one in eight of these victims were children [45]. The World Bank has estimated that the complete demining of Ukraine will cost more than $37 billion [46].

Almost one-third of the land in Ukraine is contaminated with unexploded ordnance, which includes artillery shells, grenades, mortar shells, cluster munitions, rockets, missiles, and improvised explosive devices. The failure rates among of some types of munitions can be very high, and those munitions that fail to explode initially may explode unpredictably at any time [44–48]. After the war ends, it may take up to 50 years to clear all of the landmines and unexploded ordnance [43].

### Landscape and habitat destruction

Ukraine is part of the “Green Heart of Europe”, which includes rare steppe ecosystems, coastal wetlands, alpine meadows, ancient beech forests, and extensive peatlands [49]. It is home to much of Europe’s biodiversity, including 70,000 plant, animal, and bird species, many of which are rare and endemic (found only in one place). Agricultural lands, which comprise 69% of Ukraine, hold 25% of the world’s chernozem, a rich, highly fertile type of soil [50]. Forests comprise 18% of the land.

About 30% of the country’s protected areas, covering more than 1.2 million hectares and including 23 national parks and nature preserves, have been adversely affected by military activities [51]. These habitats have been destroyed by landscape destruction (digging of trenches and fortifications, large-scale military movements, and landmines), shelling, wildfires, deforestation, and pollution. In addition to landmines and unexploded ordnance, much of the landscape is contaminated with destroyed military equipment and other remnants of war. These destructive military activities have affected the structure and function of terrestrial and aquatic ecosystems [16]. Some animals, such as the steppe eagle, black stork, brown bear, Eurasian lynx, and barn owl, are on the verge of extinction due to constant military activities in their habitats. These military activities and Russia’s occupation of territory have also prevented conservation activities.

### Other concerns

#### Radiation risks

Ukraine has four nuclear power plants, housing 15 pressurized water reactors of Russian VVER design, which prior to the 2022 invasion met 50% of domestic electricity needs. Ukraine is also home to the former Chornobyl nuclear power plant site and its contaminated exclusion zone. Other relevant radiation sites include facilities storing spent fuel from nuclear power plants and other radioactive waste, the Yuniy Komunar mine, uranium mining and reprocessing facilities, and research, medical, and industrial facilities that use nuclear sources, (such as the experimental “Neutron Source” reactor at the Kharkiv Institute of Physics and Technology, which was shelled in March and July of 2022) [52].

On March 4, 2022, the Zaporizhzhia Nuclear Power Plant (ZNPP) in southeastern Ukraine became the first operating civil nuclear power plant to ever come under armed attack. Protocol I, a 1977 amendment to the Geneva Conventions, prohibits attacks on nuclear-power generating stations, even where these stations are targets of military objectives, because such attacks may cause the release of dangerous forces and consequent morbidity and mortality among civilians [53]. The Soviet Union ratified Protocol I in 1989, but Russia revoked this ratification in 2019 [54]. Russia’s seizure and continued occupation of the ZNPP has raised domestic and international concerns about the risk that the military activities might lead to a catastrophic release of ionizing radiation. Since being occupied by Russian forces, the ZNPP has suffered from sporadic shelling, repeated disruptions of its external electrical power and threats of disruption to its water supply [53]. The International Atomic Energy Agency, which was allowed to visit the ZNPP in September 2022, has stationed representatives there to try to safeguard the site for the rest of the war.

The potential for widespread radiation release from the ZNPP, while possible, is mitigated by the following factors [55]:

- The ZNPP is designed to withstand natural and man-made hazards. Thick, steel-reinforced concrete containment buildings protect the reactor cores and are designed to keep any radioactive materials isolated from the environment.
- Unlike the Chornobyl NPP, the ZNPP reactors use the same pressurized light-water technology as nuclear reactors in the West. The spent fuel assemblies are initially stored and cooled in spent-fuel pools inside the reactor containment before being transferred outdoors to a dry spent-fuel storage facility on site.
- Nuclear power plants require back-up electricity supplies to provide cooling for the removal of decay heat produced by reactors and to maintain services, such as communications and controls. The ZNPP has four main high-voltage offsite power lines as well as back-up lines connected to a thermal power plant. In the event of complete loss of offsite power, the reactors have diesel generators that could provide power for a few weeks.
- In September 2022, all six reactors at the ZNPP were put into cold shutdown mode, in which the control rods were fully inserted into the fuel. In this mode, temperature and pressure are reduced to well below operating levels, thereby reducing the risk of a prompt radiation release [56]. A cold shutdown, however, does not eliminate the risk [53]. If cooling were disrupted for one or more of the reactors, there would be a longer period of time-- days instead of hours -- for operators to fix the problem before the cooling water in the reactor cores would start to boil away and drop below the tops of the fuel assemblies, causing the fuel to overheat and degrade.

In December 2022, two of the reactors in cold shutdown were prepared for low-power operation [56]. In this state, temperature and pressure are allowed to increase in preparation for hot standby mode, followed by zero-power operation and then low-power operation. In 2023, the shutdown state of these reactors has varied from cold to hot, depending on conflict-related disruptions [57]. Following the destruction of the Nova Kakhovka Dam in June 2023 (see below), the water levels in the reservoir that supplies the ZNPP fell. Water is needed for residual heat removal from the reactors and used-fuel ponds. Onsite back-up options are estimated to be sufficient for up to several months [58].

Even under routine conditions, during reactor shutdown operators could unleash severe accident sequences [56, 58]. Factors increasing the risk include continued armed conflict, the stress and difficult working conditions for staff members, and the threat of sabotage. In the event of a catastrophic release of radiation from the ZNPP, the extent and direction of the radioactive plume would depend on weather conditions, wind directions, and wind speed that could potentially contaminate as many as seven adjacent countries [59–61].

### The destruction of the Nova Kakhovka Dam

On June 6, 2023, an explosion breached the Nova Kakhovka Dam on the Dnipro River in southern Ukraine, releasing 19.9 billion cubic meters of water from the Kakhovka Reservoir, which flooded 77 settlements, more than 100,000 hectares (247,000 acres) of agricultural lands, nature parks, and forests downriver. While Ukraine and Russia blamed each other for the explosion, Russia was occupying the dam at the time and had the means, motive, and opportunity to destroy the dam [62]. Article 56 of Protocol I to the Geneva Conventions protects dams against attack, with extraordinarily few exceptions (even when they are targets of military objectives), if the attack “may cause the release of dangerous forces and consequent losses among the civilian population” [63].

The destruction of the Nova Kakhovka Dam has been a major humanitarian and ecological disaster. Rescuers and volunteers evacuated more than 4,000 people, but more than 50 people were killed [64]. Hundreds of thousands of people lost access to safe drinking water. Crops became waterlogged and much of the 2023 harvest has been destroyed. The dam collapse killed tens of thousands of fish as well as an estimated 20,000 animals. The flooding of nature parks and preserves killed rare flora and fauna. The Dnipro River was polluted with over 150 tons of machine oil, large amounts of organic wastes, and an unknown number of landmines that were dislodged by the flood waters. Many of pollutants, wastes, and landmines were carried downriver into the Black Sea.

The long-term environmental impacts of the destruction of the dam will likely be even more severe [65]. The Kakhovka Reservoir and reclamation system provided irrigation for the Kherson, Zaporizhzhia, and Dnipropetrovsk oblasts. With the loss of the dam, more than 1 million hectares of agricultural land in these three oblasts will be unusable for next the 3 to 5 years because of lack of a water supply [64]. The farmland that is no longer irrigated and cultivated will become more vulnerable to soil erosion and desertification. The collapse of the dam has reduced the volume of water available to the North Crimean Canal, the main source of freshwater for the Crimean Peninsula. Satellite images show that these canals are already drying up [66]. The Ukrainian Grain Council has estimated that the flood could lead to a 14% reduction in the volume of Ukraine’s grain exports. Most of this impact will be felt in low- and middle-income countries that rely on these exports. According to the Ukrainian Ministry of Energy and Natural Resources, the destruction of the dam has resulted in an estimated $3.1 billion in economic damages [11].

### Investigating environmental impacts during times of conflict

There are currently no international standards for measuring environmental impacts during war [50]. Nevertheless, several countries and international bodies, including Ukraine, the European Union, the United States, and the United Nations Development Program, have developed monitoring and documentation strategies to assess the environmental impacts of armed conflict [50, 51, 67]. Preliminary damage assessment includes determining the footprint of the war zone, identifying environmental resources at risk, identifying the pre-war baseline environment conditions, and estimating the change in resources likely affected by military activities and the value of damages to the environment [67]. Preliminary assessment focuses on material loss -- not the full impact of the war on ecosystem services. A complete assessment of the environmental impacts will be possible only after the war has ended. Data used in this preliminary assessment include a written and visual record of publicly available data from the Internet, remote sensing using real-time satellite and drone technologies, open-source information collected by civilians using photographs and eyewitness statements, and site visits with environmental sampling, when possible.

**Fig. 2:**
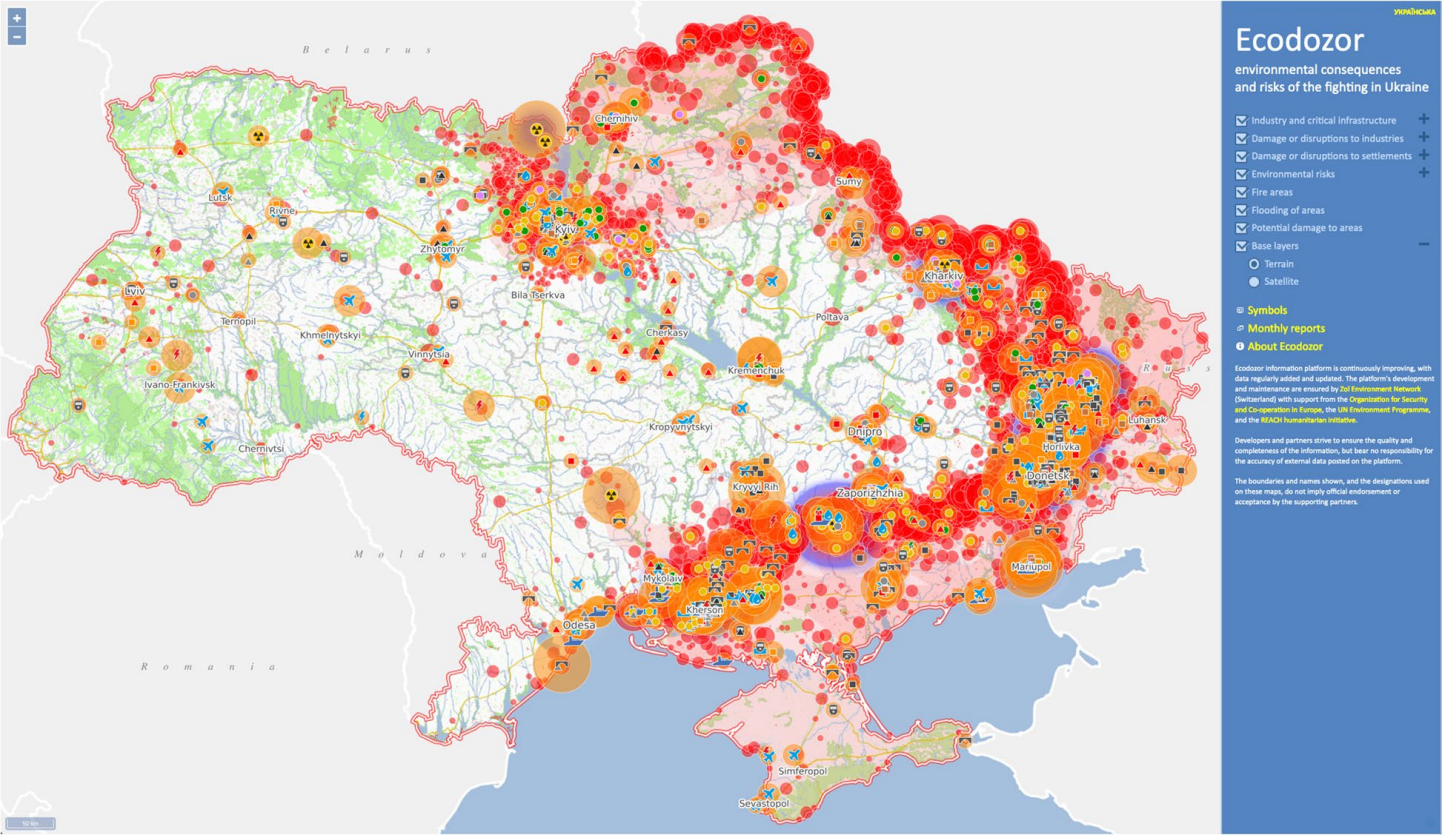
The Ecodozor Platform for mapping the environmental consequences and risks of the fighting in Ukraine. https://www.ecodozor.org/index.php?lang=en

An unprecedented volume of data about the impact of the war on the environment has been gathered by Ukrainian government agencies, civil society organizations, and international partners. These assessments will help inform remediation efforts and collect evidence needed to determine war reparations. The EcoZagroza platform has been developed by the Ukraine’s Ministry of Environmental Protection and Natural Resources [68]. The Ecodozor platform, which has been developed by the Zoï Environment Network together with the United Nations Environment Programme, the Organization for Security and Co-operation in Europe, and the REACH Humanitarian Initiative [69], is providing timely mapping of the impacts of the war on settlements, industry, and infrastructure as well as fires, floods, and sites at risk of air, water, and soil pollution (see Fig. 2). Civil society platforms include SaveEcoBot [70], Ecoaction [71], and Environment People Law [72].

### Rebuilding and reconstruction

Before the war, Ukraine’s economy was carbon-intensive, with carbon dioxide emissions per unit of gross domestic product far exceeding the world average. Other structural weaknesses in the economy included a high dependence of fossil fuel imports, monopolies in key industries, greater export of raw goods than value-added products, and low investment in modernization [73]. When Ukraine emerges from the war, it will have the opportunity to transform into a green economy, which can enhance economic prosperity while ensuring sustainability and environmental protection. The World Bank estimated that, after the first year of the war, the cost to rebuild Ukraine would have been $411 billion; this cost has increased substantially since then [74]. The highest estimated needs are in transport (22%), housing (17%), energy (11%), social protection and livelihood (10%), explosive hazard management (9%), and agriculture (7%). Ukraine’s Ministry of Environmental Protection and Natural Resources estimated that, as of June 30, 2023, the environmental damages from the war amounted to $56.4 billion [11].

Ukraine and its international partners have been planning for the rebuilding of the country, even as the war continues. Ukraine established the National Council for the Recovery from the War, as an advisory body to the President, charged with the development of the Post-War Recovery and Development Plan and a new State Agency for Reconstruction and Infrastructure Development to support reconstruction projects [75, 76]. The Ukrainian government released the first version of its 10-year national recovery plan at a heads-of-states Ukraine Recovery conference in Lugano, Italy, in July 2022. The plan proposed recovery pathways for major sectors at a projected cost of $750 billion. Major components of the plan included reconstruction and modernization of housing and infrastructure, expanding logistical and transportation facilities, achieving energy independence, and developing renewable energy. Parallel plans have been developed by the World Bank, the European Commission, the London-based Centre for Economic Policy Research, the German Marshall Fund of the United States, and other organizations [73].

The outcome of the Lugano conference was a declaration that set forth the principles for the international recovery effort:

- The overarching goal of reconstruction should be to transform Ukraine’s economy and society by modernization -- not only of its infrastructure, but also of its economic, political, and social institutions, paving the way for it to join the European Union.
- The recovery process should rebuild Ukraine in a sustainable manner, aligned with the 2030 Agenda for Sustainable Development and the 2015 Paris Agreement, integrating social, economic, and environmental dimensions, including a green energy transition.
- The keys to a green, low-carbon transformation of Ukraine’s economy are transitions to renewable energy production, decarbonizing domestic industrial processes (with an initial focus on the iron and steel industry), movement to sustainable agriculture, development of greener road infrastructure, decentralization of recovery efforts to empower cities and municipalities, and experimenting with rebuilding approaches that balance immediate needs with plans for a long-term green recovery [77–79].

Russia’s war on Ukraine has violated the United Nations Charter, which states that all members shall refrain from the use of force against the territorial integrity or political independence of any state [80]. The war has resulted in more than 500,000 military casualties, 30,000 civilian casualties, forced displacement of more than 11 million people, and more than $400 billion in estimated damage. The war has adversely impacted the global economy, decreased energy and food security, and raised the risk of nuclear war. Human, financial, and other resources that could have been used to address climate change, hunger, disease, and other societal problems have been diverted to the war.

The environment has been the silent victim of war. The immediate and short-term damages to the environment have been estimated at $56.4 billion. The long-term impacts and resulting effects on human health from pollution and damage to the natural ecosystems, can only be fully assessed once the war has ended.

A just and lasting peace requires withdrawal of Russia’s forces from the occupied territories, reparations for the damages, and rebuilding and restoration of Ukraine’s built and natural environment. When the war has ended, Ukraine will have the opportunity to rebuild in a sustainable manner by transforming to a green, low-carbon economy that is resource efficient and socially inclusive and by restoring and protecting the environment for future generations.

#### Prosecuting environmental war crimes

A body of international law proscribing military crimes against the environment has evolved since the 1970s, although enforcement is often complicated by several factors, including issues of attribution and non-signatories to various treaties. These treaties include the following [81]:

- The 1977 Additional Protocols to the Geneva Conventions: These protocols, which were adopted soon after the humanitarian crises of the Nigerian-Biafran War and the Vietnam War, described humanitarian rules for both international and non-international armed conflicts.
- The 1997 Environmental Modification Convention (ENMOD): Parties to ENMOD, which include Russia and Ukraine, prohibit the military or other hostile use of environmental manipulation that would have “widespread, long-lasting or severe effects as the means of destruction, damage or inquiry to any other State Party”.
- The 1998 Rome Statute of the International Criminal Court (ICC): Individual criminal responsibility for environmental harm was also codified under the Rome Statute. Article 8(2)(b)(iv) of the Statute states that “intentionally launching an attack in the knowledge that such an attack will cause… widespread, long-term and severe damage to the natural environment which would be clearly excessive in relation to the concrete and direct overall military advantage anticipated” is a war crime. Neither Ukraine nor Russia are signatories to the Rome Statute, but Ukraine has exercised its prerogative to accept the ICC’s jurisdiction over alleged crimes occurring on its territory.

Russia is aware of these constraints on its military actions and has been recusing itself from any treaty obligations that might govern its conduct in Ukraine. In 2019, President Vladimir Putin revoked Russia’s signature to the Additional Protocol I to the Geneva Conventions related to the protection of victims of international armed conflicts [54]. In March 2022, a few weeks after invading Ukraine, Russia was expelled from the Council of Europe -- one day after informing the organization of its intent to withdraw [82]. In February 2023, Putin signed into law a formal denouncement of the Council’s Conventions, including access to the European Court of Human Rights.

In addition to potential treaty obligations, attacks on the environment fall under the protection and authority of customary international law, including principles of humanity. The balance between humanitarian considerations and military necessity is the cornerstone of international humanitarian law. It states: “Methods and means of warfare must be employed with due regard to the protection and preservation of the natural environment” [83].

There is a strong movement to more explicitly define environmental protections under international law and, in cases of armed conflict, to build on existing *jus in bello* (law that governs the way in which warfare is conducted). In 2021, an independent panel of experts released a proposed definition of *ecocide* as “unlawful or wanton acts committed with knowledge that there is a substantial likelihood of severe and either widespread or long-term damage to the environment being caused by those acts,” with the intention of making ecocide a crime under the Rome Statute, adding to genocide, crimes against humanity, war crimes, and the crime of aggression [84].

In 2022, the International Law Commission adopted “Draft Principles on Protection of the Environment in Relation to Armed Conflicts”, which (a) prohibited “the use of methods and means of warfare that are intended, or may be expected, to cause widespread, long-term and severe damage to the environment” (Principle 13, 2[b]) and (b) stated definitively that “the law of armed conflict, including principles and rules of distinction, proportionality and precautions shall be applied to the environment, with a view to its protection” (Principle 14) [85].

Rapid development of international law often follows particularly brutal wars, such as the 1949 Geneva Conventions, which followed World War II. Given the longstanding principles of military necessity and proportionality, a strong legal argument already exists to hold Russia accountable for its military actions that have caused widespread environmental damage in Ukraine. The most likely pathway would see proceedings commence only once the war has ended. There is also an opportunity for new rules to emerge from a common conviction that increased protection is required for the only environment we have. Punishment for Russia’s lack of restraint would disincentivize aggressor states and establish better standards for the protection of human life and its dependence on a healthy environment.

## Conclusion

In addition to the enormous human costs, Russia’s war on Ukraine has had devastating impacts on the natural environment and the built environment. International law mandates that methods and means of warfare must be implemented with due regard to the protection and preservation of the natural environment. Russia’s actions will be judged not only from what is proscribed by treaty, but also on how necessary these environmentally destructive actions were from a military perspective. A just and lasting peace requires withdrawal of Russia’s forces from the occupied territories, extensive rehabilitation of individuals and communities, reparations for damages, and rebuilding and restoration of Ukraine’s natural environment and built environment.

Recognition of the environmental consequences of this war highlights the needs for investigating the environmental consequences of all armed conflicts and for implementing more effective measures to protect the environment during war. These measures need to include holding accountable those responsible for damaging the environment during war and those who instigate war.

But the only way to prevent the environmental impacts of war is to prevent war itself – by preventing conflicts from becoming violent, by addressing the underlying causes of war, and by strengthening the infrastructure for peace [86].

## Data Availability

Data cited in this study is open source.

## Abbreviations

DNB: Dinitrobenzene
2,4-DNT: 2,4- Dinitrotoluene
ENMOD: Environmental Modification Convention
HMX: Octahydro-1,3,5,7-tetranitro-1,3,5,7-tetrazocine
ICC: International Criminal Court
MEPNR: Ukraine’s Ministry of Environmental Protection and Natural Resources
NC: Nitrocellulose
NQ: Nitroguanidine
OPCN: Organization of Prohibition of Chemical Weapons
PFAS: Per- and polyfluoroalkyl substances
RDX: Hexahydro-1,3,5-trinitro- 1,3,5-triazine
tCO_2_e: Tons of carbon dioxide equivalent
Tetryl: N-methyl-N,2,4,6-tetranitroaniline
TNB: 1,3,5-Trinitrobenzene
TNT: 2,4,6-Trinitrotoluene
UN: United Nations
UNHCR: The United Nations Refugee Agency
VVER: *Vodo-vodyanoi enyergeticheskiy reactor*; translated as water-water power reactor
ZNPP: Zaporizhzhia Nuclear Power Plant
